# Topologically switchable and gated transcription machinery†

**DOI:** 10.1039/d2sc01599d

**Published:** 2022-08-18

**Authors:** Pu Zhang, Amit Fischer, Yu Ouyang, Yang Sung Sohn, Rachel Nechushtai, Junji Zhang, He Tian, Chunhai Fan, Itamar Willner

**Affiliations:** Institute of Chemistry, Center for Nanoscience and Nanotechnology, The Hebrew University of Jerusalem Jerusalem 91904 Israel itamar.willner@mail.huji.ac.il; Institute of Life Science, The Hebrew University of Jerusalem Jerusalem 91904 Israel; Key Laboratory for Advanced Materials, School of Chemistry and Molecular Engineering, East China University of Science and Technology Shanghai China; School of Chemistry and Chemical Engineering, Frontiers Science Center for Transformative Molecules, National Center for Translational Medicine, Shanghai Jiao Tong University 200240 Shanghai China

## Abstract

Topological barriers control in nature the transcription machinery, thereby perturbing gene expression. Here we introduce synthetically designed DNA templates that include built-in topological barriers for switchable, triggered-controlled transcription of RNA aptamers. This is exemplified with the design of transcription templates that include reversible and switchable topological barriers consisting of a Sr^2+^-ion-stabilized G-quadruplex and its separation by kryptofix [2.2.2], KP, for the switchable transcription of the malachite green (MG) RNA aptamer, the T-A·T triplex barrier being separated by a fuel-strand for the cyclic triggered transcription of the 3,5-difluoro-4-hydroxybenzylidene imidazolinone (DFHBI)-binding aptamer, and the use of a photoactivated *cis*/*trans* azobenzene-modified nucleic acid barrier for the switchable “ON”/“OFF” transcription of the MG RNA aptamer. By applying a mixture of topologically triggered templates consisting of the photoresponsive barrier and the T-A·T triplex barrier, the gated transcription of the MG aptamer or the DFHBI-binding aptamer is demonstrated. In addition, a Sr^2+^-ion/KP topologically triggered DNA tetrahedra promoter-transcription scaffold, for the replication of the MG RNA aptamer, and T7 RNA polymerase are integrated into DNA-based carboxymethyl cellulose hydrogel microcapsules acting as cell-like assemblies. The switchable, reversible transcription of the MG RNA aptamer in a cell-like containment is introduced.

## Abstract 2

Three different topological barriers to switch transcription machineries were introduced including Sr^2+^-ion stabilized G-quadruplex units, T-A·T triplex structures and photoisomerizable azobenzene-nucleic acid blockers.

## Graphical abstract

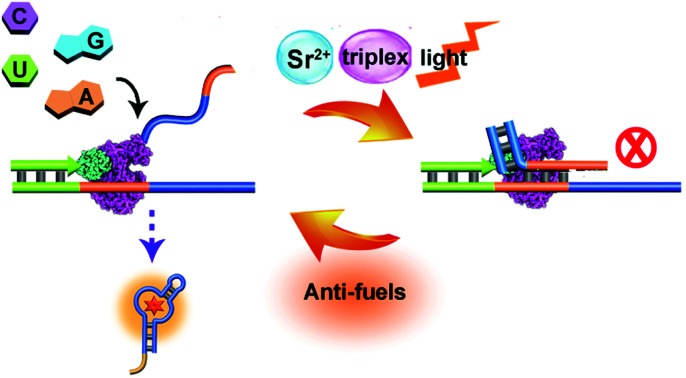

## Introduction

Topological barriers associated with condensed chromatin regulate the transcription of mRNA through the suppression of the accessibility of transcription factors or RNA polymerase to the transcription machinery.^1–5^ The development of *in vitro* programmed reversible topologically regulated transcription machinery could lead to triggered, dose-controlled gene expression for medical applications.^6^ The base sequence encoded in nucleic acids provides a means to reversibly modulate and switch the topologies of DNA nanostructures by auxiliary triggers. For example, ion-induced formation of G-quadruplexes and their separation in the presence of crown ethers,^7,8^ the stabilization of T–T and C–C mismatched duplexes by metal ions, such as Hg^2+^ or Ag^+^, and their separation by metal-ion binding ligands,^9–11^ the reversible pH-stimulated formation of i-motif structures,^12^ the formation of T-A·T triplex structures and their disassembly by fuel strand-driven displacement processes,^13^ and the photochemical stabilization/destabilization of DNA duplexes by photoisomerizable intercalators, such as azobenzene units,^14,15^ provide a rich “tool-box” of reconfigurable DNA topologies. Indeed, these switchable DNA structures were applied to assemble DNA switches^16^ and DNA machines,^17–19^ such as tweezers,^20,21^ walkers^22–25^ or nanocarriers,^26–28^ to design “smart” materials acting as gated drug carriers for controlled drug release, such as SiO_2_ nanoparticles,^29–31^ metal–organic framework nanoparticles^32–34^ or microcapsules,^35,36^ and to prepare stimuli-responsive hydrogels exhibiting switchable stiffness properties for shape-memory,^37,38^ self-healing,^39,40^ controlled drug-release^41^ and mechanical actuating applications.^42^

The reconfigurable DNA structures allow, in principle, the engineering of DNA templates that include topological barriers for operating reversible and cyclic transcription machineries. Here, we report on the design of switchable transcription machineries driven by Sr^2+^-ion stabilized G-quadruplex/kryptofix [2.2.2] (KP), T-A·T triplex displacement strands, and photoresponsive *trans*/*cis* azobenzene intercalator units. By mixing two scaffolds of topologically reconfigurable transcription machineries composed of *trans*-azobenzene-duplex bridged blocker topologies and the T-A·T triplex, the programmed and reversible gated operation of transcription machineries synthesizing target RNAs is demonstrated. It should be noted that in a recent report,^43^ the triggered operation of a topologically hindered transcription machinery was reported. In this system, the DNA template was caged in a DNA tetrahedral nanostructure. The separation of the tetrahedral nanostructure by a fuel strand yielded an active template that facilitated the RNA polymerase/NTPs replication of RNAs. This system is, however, not reversible and could not be cycled across “ON”–“OFF” states. In addition, we demonstrate the Sr^2+^-ions/KP switchable transcription machinery in hydrogel microcapsule cell-like containments as a model system for operating a triggered transcription process in a cell-like microenvironment.^44–46^

## Results and discussion

**Fig. 1:**
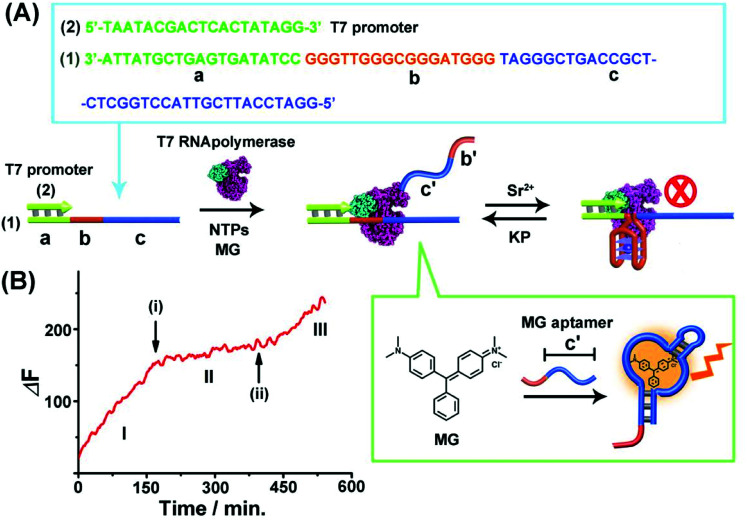
(A) Schematic of Sr^2+^-ions/kryptofix [2.2.2], KP, topologically switchable transcription of the MG aptamer. (B) Time-dependent switchable fluorescence changes upon the transcription of the MG aptamer in the presence of T7 RNA polymerase, NTPs and MG. (I) In the absence of Sr^2+^ ions. (II) At time marked (i), Sr^2+^ ions, 0.02 M, are added. (III) At time marked (ii), KP, 0.06 M, is added. The experimental results were reproduced by an *N* = 5 experiment and the results demonstrated deviations corresponding to ±5%.

Fig. 1(A) depicts the reversible Sr^2+^-ion stabilized G-quadruplex/kryptofix [2.2.2] (KP)-induced switchable transcription of the malachite green (MG) RNA aptamer. The association of MG with its aptamer switches-ON the fluorescence of MG (*λ*_em_ = 665 nm) and, thus, the transcription process is followed by the temporal fluorescence changes of the transcribed MG/aptamer complex. The transcription module includes a DNA duplex template consisting of scaffold (1) and the T7 promoter (2). The strand (1) includes three domains: (a) the T7 promoter binding sequence, (b) the guanosine (G)-rich sequence that self-assembles under appropriate conditions to the G-quadruplex unit, and the domain (c) that is complementary to the MG aptamer sequence. Subjecting the template (1)/(2) to T7 RNA polymerase and the NTPs mixture, in the presence of MG, results in the triggered transcription machinery that replicates the domains (b) and (c) to form the RNA strand (b')/(c'), where (c') corresponds to the MG aptamer. The generation of the MG aptamer is followed by the time-dependent fluorescence changes of the formation of the MG/aptamer complex.^47^Fig. 1(B), part I depicts the time-dependent fluorescence changes upon the transcription of the MG aptamer. The addition of Sr^2+^ ions to the reaction machinery, time marked (i), reconfigures domain (b) into the Sr^2+^-ion stabilized G-quadruplex structure. The formation of the G-quadruplex topological barrier blocks the transcription process and inhibits the formation of the aptamer, Fig. 1(A) and (B), part II. At time marked (ii), KP is added to the system, resulting in the separation of the Sr^2+^-ion stabilized G-quadruplex blocker units, and the reactivation of the transcription of the MG aptamer, Fig. 1(A) and (B), part III. For further experiments supporting the formation of the Sr^2+^-ion stabilized G-quadruplex, see ESI, Fig. S1 and S2† and accompanying discussion. It should be noted that in contrast to the common application of a promoter/duplex template configuration of the transcription machinery, we apply throughout the study promoter/single strand templates as transcription machineries. We find that the promoter/duplex template assemblies lead to lower (incomplete) switching efficiencies upon the triggered topological reconfiguration of the template strand, as compared to the switching efficiency of the promoter/single strand configuration (see Fig. S3† ESI and accompanying discussion). This is due to the fact that the triggered topological reconfiguration of the promoter/double stranded configuration includes always a residual content of a non-reconfigured double-stranded template that generates the transcription product. In addition, we note that for the G-quadruplex/KP reversible modulated ON/OFF switchable synthesis of the MG aptamer, we employed the Sr^2+^-ion stabilized G-quadruplex (rather than the common K^+^-ion stabilized/crown ether switching system). This is due to the fact that the K^+^-ion stabilized G-quadruplex/crown ether system was found to quench the fluorescence of the MG/aptamer complex, thus perturbing the readout signal of the respective transcription machinery.

**Fig. 2:**
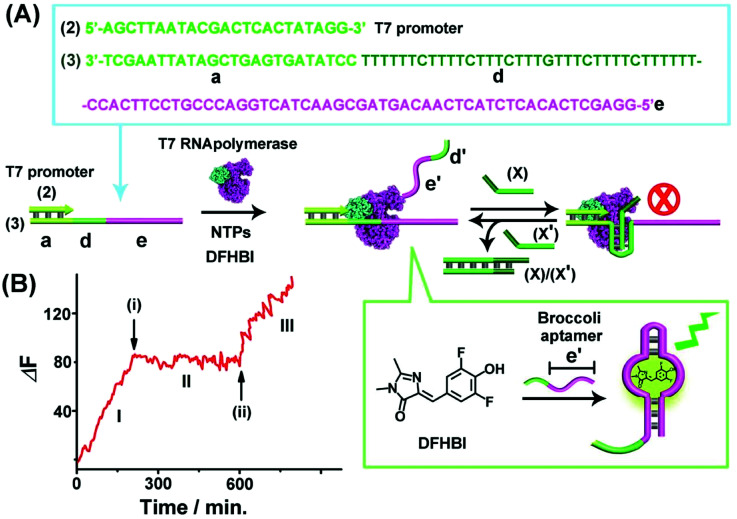
(A) Schematic of T-A·T triplex topologically switchable transcription of the DFHBI-binding aptamer in the presence of T7 RNA polymerase, NTPs and DFHBI. The strand (X) yields the triplex structure to switch-OFF the transcription process, whereas the displacement of (X) by the strand (X′) switches-ON the transcription machinery. (B) Time-dependent fluorescence changes upon the switchable transcription of the DFHBI/aptamer complex by the transcription machinery. (I) In the absence of strand (X). (II) In the presence of strand (X), 10 μM, introduced at time marked (i). (III) At time marked (ii), (X′), 15 μM, is added to the system. The experimental results were reproduced by an *N* = 5 experiment and the results demonstrated deviations corresponding to ±5%.

The second switchable transcription process included the T-A·T triplex as the topological blocker unit. This is exemplified in Fig. 2(A) with the switchable transcription of the Broccoli aptamer that binds 3,5-difluoro-4-hydroxybenzylidene imidazolinone, DFHBI, to yield a fluorescent DFHBI/aptamer complex (*λ*_em_ = 500 nm).^48^ The transcription machinery is composed of the template (2)/(3) where the scaffold (3) includes the domains (a), (d) and (e). Domain (a) corresponds to the T7 promoter (2) binding sites, domain (d) consists of a thymine (T)-rich sequence, being capable of forming a T-A·T triplex structure in the presence of an appropriate trigger, and domain (e) is complementary to the broccoli aptamer sequence. Subjecting the template (2)/(3) to a T7 RNA polymerase and NTPs mixture, in the presence of the DFHBI ligand, activates the transcription machinery and the synthesis of the broccoli RNA aptamer that binds DFHBI. The fluorescence changes of the resulting DFHBI/aptamer complex transduce the transcription process. Fig. 2(B), part I, shows the time-dependent fluorescence changes upon the formation of the DFHBI/aptamer complex. At time marked (i), the strand (X) is added to the transcription machinery. The strand (X) reconfigures domain (d) of scaffold (3) into a T-A·T triplex blocker that switches-OFF the transcription process, Fig. 2(A) and (B), part II. The blocker strand is engineered, however, to include a toehold tether, that upon further addition of the strand (X′) displaces the blocker unit, to yield the duplex (X)/(X′), resulting in the uncaging of the topological barrier and the reactivation of the transcription process generating the DFHBI binding aptamer, Fig. 2(B), part III, time marked (ii). Moreover, an alternative template (2)/(4) consisting of a T-A·T triplex, switchable transcription machinery for synthesizing the MG aptamer was engineered and the results of this system are presented in Fig. S4.† In this system, the scaffold (4) includes the domains (a), (d) and (c), where (a) provides the promoter binding site, (d) corresponds to the T-rich sequence that forms, in the presence of the triggering strand (X), the T-A·T triplex, and the domain (c) is complementary to the MG aptamer sequence. Fig. S4(A) and (B)† demonstrate the T-A·T triplex-based switchable transcription of the MG aptamer. That is, the reversible topological T-A·T triplex blocker allows the switchable transcription of the DFHBI-binding RNA aptamer or the MG RNA aptamer. It should be noted, however, that in contrast to the triplex blocker that allows the transcription of the DFHBI or the MG aptamer by appropriate design of the template machinery, the Sr^2+^-ion triggered G-quadruplex blocker unit could not be applied to design a DFHBI-binding aptamer transcription machinery. The DFHBI-binding aptamer includes a G-quadruplex domain that associates with the Sr^2+^ ions and this perturbs the fluorescence features of DFHBI, thus disturbing the readout of the potentially G-quadruplex switching of the DFHBI transcription machinery.

**Fig. 3:**
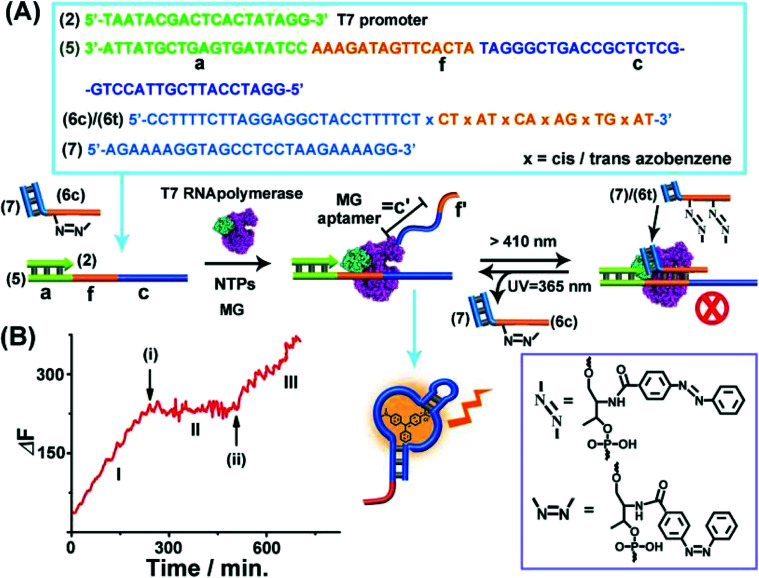
(A) Light-induced reversible switching of the MG aptamer transcription machinery operating in the presence of the (2)/(5) template, *cis*-azobenzene-modified (6c)/(7) photoactive units, T7 RNA polymerase, NTPs and MG. Photochemical isomerization of (6c)/(7) to the *trans*-state, (6t)/(7), *λ* > 410 nm, blocks the transcription machinery, whereas the reverse photoisomerization of (6t)/(7) to (6c)/(7), *λ* = 365 nm, reactivates the transcription machinery. (B) Time-dependent fluorescence changes upon the light-induced switchable transcription of the MG aptamer. (I) Transcription machinery in the presence of (6c)/(7), 5 μM. (II) In the presence of the (6t)/(7) module generated at time (i) by illumination of the system, *λ* > 410 nm, for 10 minutes, photoisomerizing (6c)/(7) to (6t)/(7). (III) At time marked (ii), in the presence of (6c)/(7) formed by irradiation of the system *λ* = 365 nm for 10 minutes photoisomerizing the (6t)/(7) to (6c)/(7). The experimental results were reproduced by an *N* = 5 experiment and the results demonstrated deviations corresponding to ±5%.

The third topologically triggered transcription machinery involved light-induced control over the transcription machinery using *trans*/*cis* azobenzene photoisomerizable blocker units. The *trans* azobenzene units modifying the nucleic acid strand intercalate into duplex nucleic acid structures, thereby cooperatively stabilizing the duplex DNA structure, whereas the photoisomerized *cis*-azobenzene tether forms a steric hindrance between the duplex nucleic acid structure thereby destabilizing the duplex structures. Thus, by the appropriate design of a photoisomerizable auxiliary azobenzene-functionalized strand, the light-induced switchable transcription machinery is realized. Fig. 3(A) depicts the principle to reversibly modulate the switchable transcription machinery by light. The template composed of the duplex (2)/(5) and the auxiliary duplex consisting of (7) and the photoresponsive *cis*-azobenzene functionalized strand (6c) acts, in the presence of T7 RNA polymerase and NTPs, as the photo-triggered transcription machinery. (The strand (6c) is composed of two domains, one domain hybridizes with (7), and the other domain contains the photoisomerized *cis*-azobenzene units). The strand (5) being a part of the transcription machinery includes the domains (a), (f) and (c). Domain (a) provides the T7 promoter with a binding site, the domain (f) is engineered to interact with the photoresponsive blocker unit (6)/(7), and domain (c) is complementary to the MG aptamer sequence. The *cis*-azobenzene (6c)/(7) auxiliary unit does not interact with domain (f) of the transcription machinery; thus subjecting the transcription machinery to a T7 RNA polymerase and NTPs mixture, in the presence of MG, triggers-ON the transcription of the MG aptamer, (c′), which is followed by the fluorescence changes of the MG/aptamer complex. Fig. 3(B), part I shows the time-dependent fluorescence changes, resulting in the formation of the fluorescent MG/aptamer complex upon the transcription of the MG aptamer. At the time marked with an arrow (i), the system is illuminated at *λ* > 410 nm, leading to the photoisomerization of the *cis*-azobenzene-functionalized module (6c)/(7) to the *trans*-state, (6t)/(7). This results in the hybridization of the *trans*-azobenzene topological unit to the domain (f) of template (5), forming a topological barrier that switches-OFF the transcription process, Fig. 3(A), and the switched-OFF transcription process is demonstrated in Fig. 3(B), part II. Further irradiation of the system, *λ* = 365 nm, photoisomerizes the *trans*-azobenzene blocker element into the *cis*-state (6c)/(7), resulting in the separation of the *cis*-azobenzene (6c)/(7) unit from the template and the reactivation of the transcription process, Fig. 3(A) and (B), arrow (ii), part III. It should be noted that the use of the duplex blocker unit (6t)/(7) is important to switch-OFF the transcription process. Applying (6t) as a single strand yielding the duplex between (6t) and domain (f) of (5) and the single toehold tether linked to (6t) still allows the T7 RNA polymerase to proceed. Presumably,^49,50^ the rigid duplex structure of (6t)/(7) introduces a bulky topological barrier that perturbs and inhibits the transcription of the scaffold.

**Fig. 4:**
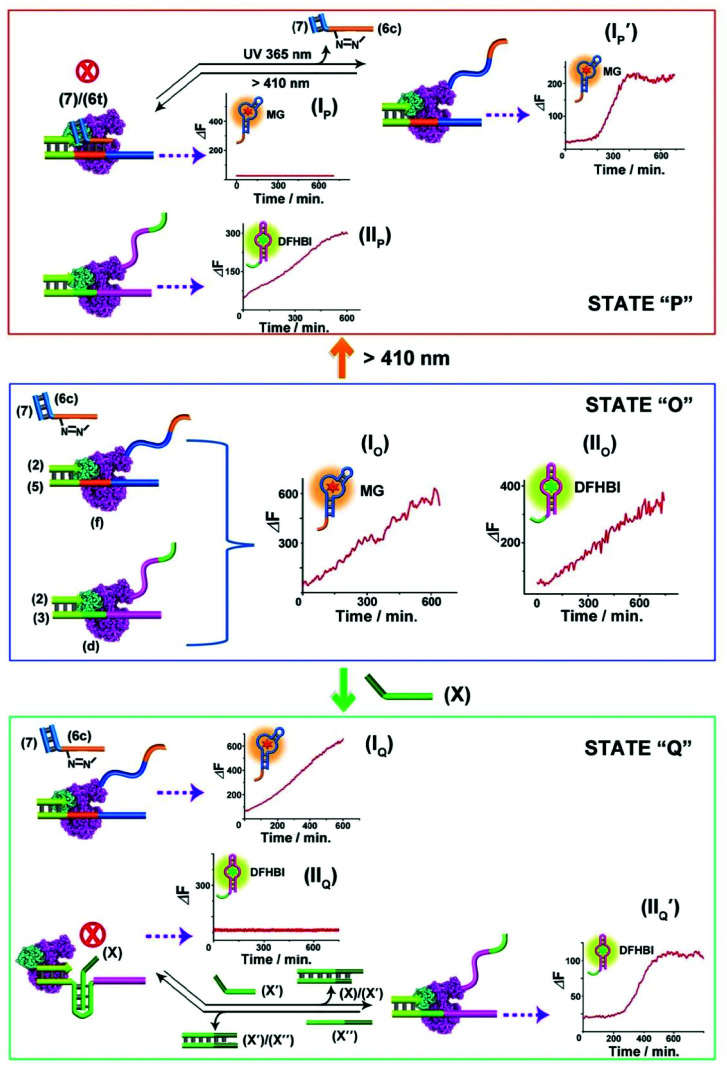
Topologically gated transcription machineries: the reaction module in state “O” includes two scaffolds of topologically responsive transcription machineries consisting of the (2)/(5) template and (6c)/(7) photoresponsive machinery and the triplex-responsive (2)/(3) template transcription machinery. Treatment of the module in state “O” with T7 RNA polymerase and NTPs leads to the parallel operation of the two scaffolds of transcription machineries to yield the MG aptamer, panel I_O_, and the DFHBI-binding aptamer, panel II_O_. Subjecting state “O” to visible light, *λ* > 410 nm, yields state “P” where the MG transcription is blocked, panel I_P_, leading to the gated transcription of the DFHBI-binding aptamer, panel II_P_. Treatment of the module state “O” with the strand (X) leads to the topologically triplex-inhibited DFHBI-binding aptamer transcription machinery, panel II_O_, state “Q”, and to the gated transcription of the MG aptamer, panel I_O_. Each of the gated machinery components can be recovered to the non-gated transcription module state “O”, by illumination at *λ* = 365 nm, panel I_P_′, or the dissociation of the triplex barrier by the counter strand (X′′), panel II_Q_′ respectively. The experimental results were reproduced by an *N* = 3 experiment and the results demonstrated deviations corresponding to ±5%.

The successful design of topologically switchable transcription machineries using different triggers to yield two different aptamers (the MG and DFHBI-binding aptamer) suggested that gated transcription machinery guiding the selective synthesis of target RNAs could be designed. Fig. 4 presents the gated operation of two scaffolds of transcription machineries synthesizing the MG and DFHBI-binding aptamers, using the photoresponsive *trans*-azobenzene blocker and the T-A·T triplex blocker as gating units. The system consists of a mixture of the two scaffolds of transcription machineries, state “O” composed of the (2)/(5) and (6c)/(7) photoresponsive machinery that transcribes the MG aptamer and the T-A·T triplex-responsive template (2)/(3) that transcribes the DFHBI-binding aptamer. Subjecting the mixture to a T7 RNA polymerase and NTPs mixture, in the presence of MG and DFHBI, triggers the two scaffolds of transcription machineries to yield the MG aptamer and DFHBI-binding aptamer, panels I_O_ and II_O_, respectively. The irradiation of state “O”, *λ* > 410 nm, transforms the (6c)/(7) module into the *trans*-azobenzene module (6t)/(7) that hybridizes with domain (f) of template (5), resulting in the blocking of the photoresponsive machinery, state “P”, panel I_P_, while the triplex responsive machinery is unaffected, panel II_P_. That is, the photochemical transition of state “O” to state “P” gates the transcription machinery by blocking the transcription of the MG aptamer, panel I_P_, while allowing the transcription of the DFHBI-binding aptamer, Fig. 4, panel II_P_. The caged inactive MG aptamer transcription machinery can be, however, switched between “ON” and “OFF” states by the cyclic photoinduced isomerization of the photoresponsive units between the (6c)/(7) (*λ* = 365 nm) and the (6t)/(7) (*λ* > 410 nm) units, respectively, panel I_P_′. Similarly, state “O” can be triggered by the strand (X) that transforms state “O” into state “Q”, where the domain (d) of strand (3) is reconfigured into the topological T-A·T triplex blocked the transcription machinery. That is, subjecting strand (X) to state “O” results in the transcription of the MG aptamer, by the photoresponsive machinery panel I_Q_, while the transcription of the DFHBI-binding aptamer is blocked, panel II_Q_. The T-A·T triplex topologically blocked machinery can be, however, reversibly triggered between “ON” and “OFF” states. The treatment of the system with the anti-blocker strand, (X′), and the subsequent re-treatment of the switched-ON transcription machinery of the DFHBI-binding aptamer with the strand (X′′) result in the cyclic activation/deactivation of the DFHBI-binding aptamer formation, panel II_Q_′.

**Fig. 5:**
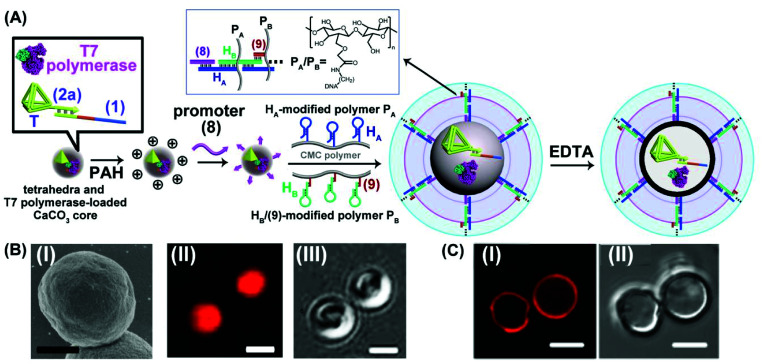
(A) Schematic integration of the DNA tetrahedra (T)-modified template (1)/T-(2a) corresponding to the MG aptamer transcription machinery and T7 RNA polymerase into a DNA-crosslinked CMC microcapsular containment. (B) Panel I-SEM image of the CaCO_3_ core particle impregnated with T7 RNA polymerase and the (1)/T-(2a) transcription template, coated with the DNA-crosslinked CMC hydrogel (scale bar: 1 μm). Panel II and III-confocal fluorescence microscopy image and bright field image corresponding to the microcapsules loaded with a fluorophore Cy5-labeled tetrahedra-modified template (1)/T-(2a) and T7 RNA polymerase before etching (scale bar: 2 μm). (C) Confocal fluorescence microscopy image, panel I, and bright field image, panel II, of the etched microcapsules loaded with fluorophore Cy5-labeled tetrahedra-modified template (1)/T-(2a) and T7 RNA polymerase (scale bar: 2 μm).

The different scaffolds of topologically switchable transcription machineries are operated in a homogeneous buffer solution. Their operation in a cell-like containment could provide a further complex design of transcription processes mimicking native systems. Different cell-like containments acting as cell-like containments were developed in the past few years. These include liposomes,^51,52^ polymersomes,^53–55^ proteinosomes,^56–59^ colloidosomes^60^ and coacervate microdroplets.^61^ Our laboratory developed in the past few years a versatile method to design nucleic acid-based hydrogel microcapsules, and particularly stimuli-responsive microcapsules.^62^ These microcapsules were loaded with drugs, nanoparticles or proteins and used as functional carriers for the release of the loads. The preparation of the microcapsules involves the impregnation of CaCO_3_ microparticles with the loads, the functionalization of the particles with promoter-nucleic acid tethers, and the interaction of the modified microparticles with two polymer chains functionalized with two types of pre-engineered hairpins H_A_ and H_B_. Subjecting the promoter modified microparticles to the hairpin-functionalized polymer chains triggered the inter-polymer hybridization chain reaction (HCR) resulting in a hydrogel coating crosslinked by the inter-hybridized H_A_/H_B_ duplexes. The subsequent etching of the CaCO_3_ core templates with ethylenediaminetetraacetic acid, EDTA, yielded substrate-loaded microcapsules. We find that besides using microcapsules as drug carriers, they can be applied as cell-mimicking containments. By appropriate engineering of the hydrogel coating, molecular entities with a molecular weight *ca.* ≥5 kDa are retained in the cell-like containment with no leakage to the bulk solution, yet a molecular or ionic substrate of a lower molecular weight, ≤5 kDa, can diffuse in-and-out across the microcapsular boundaries.^35^ We find that the nucleic acid-based hydrogel microcapsules reveal beneficial properties as compared to other protocell containments. The microcapsules revealed high-loading capacities, rapid exchange of low-molecular-weight agents, allowing effective communication with the bulk surrounding solution, low leakage properties towards constituents exhibiting molecular weight ≤ *ca.* 5 kD, and relatively high stabilities.^63^ Accordingly, we designed a cell-like containment in which the topologically triggered switchable transcription of the MG aptamer is demonstrated, Fig. 5. The assembly of the microcapsules loaded with MG aptamer transcription machinery is depicted in Fig. 5(A). The duplex consisting of the template scaffold (1) hybridized with the nucleic acid tetrahedra (T)-functionalized T7 promoter (2a) was deposited together with T7 RNA polymerase on CaCO_3_ microparticles. (The tetrahedral units were tethered to the T7 promoter to ensure the non-leakable containers of the transcription template to the resulting microcapsules). The loaded CaCO_3_ particles were, then, coated with polyallylamine hydrochloride, PAH, modified with the nucleic acid promoter (8), and the particles were subjected to two polymer chains P_A_ and P_B_ consisting of carboxymethyl cellulose, CMC, functionalized with the hairpins H_A_/H_B_. The hairpin H_A_ was tethered to polymer P_A_, and hairpin H_B_ was anchored through hybridization with a short tether (9) to polymer P_B_. (The specific anchoring of H_B_ with (9) is essential for directionality reasons dictating the inter-hybridization of H_A_/H_B_). Under these conditions, the promoter strands (8), functionalizing the particles, trigger the HCR process, where (8) opens hairpin H_A_, and opened H_A_, opens hairpin H_B_, and *vica versa*, resulting in a CMC hydrogel coating of the polymer chains crosslinked by the duplexes comprising inter-cross opened hairpins H_A_/H_B_. The subsequent etching of the CaCO_3_ cores with EDTA resulted in hydrogel-stabilized microcapsules loaded with the tetrahedra-modified (1)/(2a) template, (1)/T-(2a), and T7 RNA polymerase. Fig. 5(B), panel I, shows the SEM image of the T7 RNA polymerase and (1)/T-(2a) loaded CaCO_3_ microparticles coated with the CMC hydrogel before etching. For comparison, the bright field and fluorescence confocal microscopy images of the hydrogel coated CaCO_3_ particles loaded with T7 RNA polymerase and (1)/T-(2a) prior to etching are provided in panel II and panel III. The fluorescence and bright-field images of the EDTA etched microcapsules are shown in Fig. 5(C) panels I and II. It should be noted that a fluorophore (Cy5) was used to label (1)/T-(2a) for the fluorescence characterization of the microcapsules. Spherical microcapsules with *ca.* 2.5 μm diameter are formed. The loadings of the microcapsules with the Cy5-labeled (1)/T-(2a) scaffold was estimated to be *ca.* 2.4 × 10^−10^ moles in the overall volume of capsules (or 2.6 × 10^−16^ moles per capsule) and of the T7 RNA polymerase 1.11 × 10^−11^ moles in the overall mixture of capsules (or 1.23 × 10^−17^ moles per capsule). The loadings of the microcapsules with fluorescent (1)/T-(2a) was evaluated by following the fluorescence intensities of the resulting microcapsules and concomitantly evaluating the residual fluorescent (1)/T-(2a) in the washing solutions upon the preparation of the microcapsules. The loadings of T7 RNA polymerase were estimated by following the absorbance feature of the protein. (For details, see ESI, Fig. S5†). The resulting microcapsules were stable for at least three days, and no leakage from the microcapsules was detected within this time-interval, Fig. S6.† The intact structure of the tetrahedra-modified strand (2a), T-(2a), was confirmed by electrophoretic characterization, Fig. S7,† and the intact structure of (1)/T-(2a), under the conditions applied to prepare the microcapsules (Fig. S8†), and the activity of the (1)/T-(2a) and T7 RNA polymerase, subjected to the conditions to prepare the microcapsule (Fig. S9†) were evaluated by independent experiments. While it is impossible to monitor either the structure of the (1)/T-(2a) in the microcapsules or the activity of T7 RNA polymerase in the microcapsules, we subjected CaCO_3_ microparticles to independent impregnation with the (1)/T-(2a) template or with the T7 RNA polymerase. The impregnated microparticles were then subjected to the EDTA etching process under identical conditions used to prepare the microcapsules, and the resulting (1)/T-(2a) solution was analyzed by electrophoresis and compared to a non-etched (1)/T-(2a) solution, Fig. S8† and accompanying discussion. The results demonstrate that the deposition of (1)/T-(2a) on the CaCO_3_ core and the EDTA etching had no effect on the intact structure of (1)/T-(2a). In addition, the activity of the T7 RNA polymerase subjected to the deposition onto the CaCO_3_ particles and the subsequent etching by EDTA was compared to the parent T7 RNA polymerase activity by comparing the transcription rates of the MG RNA aptamer by the two enzyme systems, Fig. S9.† We find that the T7 RNA polymerase retained 70% of the activity of the parent enzyme after the CaCO_3_ impregnation/EDTA etching.

**Fig. 6:**
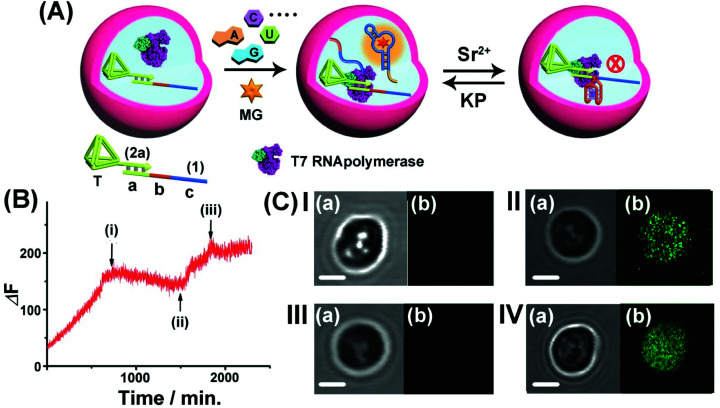
(A) Switchable Sr^2+^-ion/KP-stimulated transcription of the MG aptamer in the microcapsule assembly. The loaded microcapsules are suspended in a solution containing a MG and NTPs mixture and the Sr^2+^ ions or KP are alternately added to the exterior solution. (B) Time-dependent fluorescence changes of the suspension of the microcapsules upon the dynamic switchable transcription process. The reaction is initiated by adding to the microcapsule suspension NTPs and MG. At point (i), Sr^2+^ ions, 0.02 M, are added to the system. At point (ii), KP, 0.06 M, is added to the system. At point (iii), Sr^2+^ ions, 0.08 M, are re-added to the system. (C) Confocal microscopy images probing the switchable transcription of the MG aptamer in the microcapsules: Panel I-the microcapsules loaded with the (1)/T-(2a) template and T7 RNA polymerase in the presence of auxiliary NTPs and MG, at *t* = 0: (a) bright field (b) fluorescence image (no fluorescence). Panel II-(a) bright-field image, after the addition of MG and NTPs and allowing the transcription process to proceed for 24 hours. (b) Fluorescence image (fluorescence of the MG/aptamer complex is switched-ON). Panel III-microcapsule treated with Sr^2+^ ions, 0.01 M, and NTPs for a time interval of 24 hours. (a) Bright-field image. (b) Fluorescence image (no fluorescence is observed). Panel IV-microcapsules treated with Sr^2+^ ions 0.01 M for 24 hours, and subsequently subjected to KP 0.1 M and allowed to react for 24 hours: (a) bright-field image. (b) Fluorescence image (fluorescence in the microcapsules is switched-ON) (scale bar: 1 μm). The experimental results were reproduced by an *N* = 3 experiment and the results demonstrated deviations corresponding to ±5%.

Realizing that the T7 RNA polymerase preserves *ca.* 70% of its activity and the template (1)/T-(2a) retains its intact structure in the microcapsule, cell-like containment and the permeability features of NTPs, MG, Sr^2+^ ions and KP through the microcapsule boundaries, we applied the system as a functional topologically switchable transcription machinery of the MG RNA aptamer in a cell-like containment assembly, Fig. 6. (For the operation of the Sr^2+^-ion/KP-switchable tetrahedra (2a)/(1) transcription machinery in a homogeneous buffer solution, see Fig. S10† and accompanying discussion). The scaffold (1) consists of the three domains (a), (b) and (c), where the sequence (a) corresponds to the T7 promoter binding site, domain (b) includes a guanosine (G)-rich sequence that reveals, in the presence of Sr^2+^ ions and KP, reversible reconfigurable formation and separation of the G-quadruplex structure, and domain (c) consists of a complementary sequence to the MG RNA aptamer. The treatment of the microcapsules with NTPs and MG triggers-ON the intracapsular transcription of the MG RNA aptamer. Subjecting the microcapsules to Sr^2+^ ions is, then, anticipated to reconfigure domain (b) of (1) into the Sr^2+^-ion stabilized G-quadruplex unit that acts as a topological barrier for the transcription process. Under these conditions, the transcription of the MG aptamer is blocked. Further addition of KP to the system separates the G-quadruplex, resulting in the reactivation of the transcription process in the microcapsule. Thus, switchable intramicrocapsule operation of the topologically triggered transcription proceeds in the system. The switchable intramicrocapsule operation of Sr^2+^-ions/KP-stimulated “ON”/“OFF” transcription process is followed by the time-dependent fluorescence changes of the MG/aptamer complex in the presence of NTPs and MG, and demonstrated in Fig. 6(B). At the time (i), marked with an arrow, Sr^2+^ ions are added to the system. This reconfigures domain (b), of scaffold (1), into a Sr^2+^-ion stabilized G-quadruplex barrier that blocks the transcription process. At time-interval marked (ii), KP is added to the system. The permeation of KP into the microcapsule separates the Sr^2+^-ion stabilized G-quadruplex, resulting in the reactivation of the transcription process. At time-interval marked (iii), Sr^2+^ ions were re-added to the system, and re-blocked the transcription process in microcapsules. It should be noted that the switchable fluorescence changes observed in the microcapsules show noisy fluorescence intensities, as compared to the fluorescence data recorded for an analogous homogeneous buffer system. This is attributed to the fact that microcapsular hydrogel containments represent a microheterogeneous environment that reveals light-scattering that perturbs the fluorescence intensities. Furthermore, the relatively low number of microcapsules used in the total reaction volume, required the operation of the fluorescence spectrophotometer at enhanced sensitivities that resulted in further background noise. The switchable intracapsular transcription of the MG aptamer was further supported by bright field and confocal fluorescence microscopy experiments, Fig. 6(C). Panel I presents the bright field (a) and confocal fluorescence microscopy (b) images of the same domain of the microcapsules loaded with the (1)/T-(2a) template and T7 RNA polymerase in the presence of auxiliary NTPs and MG, at *t* = 0. Only the bright field image of the microcapsule is visible. Panel II depicts the bright field (a) and confocal fluorescence microscopy (b) images of the microcapsules loaded with the (1)/T-(2a) template and T7 RNA polymerase, after a time interval of 24 hours. The green fluorescence of the transcribed MG aptamer loaded microcapsule is observed, indicating the successful transcription of the MG aptamer. Panel III depicts the experimental results corresponding to microcapsules loaded with the (1)/T-(2a) template and T7 RNA polymerase subjected to NTPs, MG and Sr^2+^ ions, for a time interval of 24 hours. While the bright field image of the capsule is visible (a), no fluorescence of the MG aptamer loaded capsules is observed (b), consistent with the Sr^2+^-ion stabilized G-quadruplex topological inhibition of the transcription process. Panel IV depicts the images generated upon treatment of the switched-OFF microcapsule system shown in panel III with KP for a time interval of 24 hours. In addition to the bright field image of the microcapsule (a), the green fluorescence of the MG/aptamer complex formed in the microcapsule as a result of switched-ON transcription of the MG aptamer is observed (b). The result is consistent with the KP stimulated separation of the Sr^2+^-ion stabilized G-quadruplex topological barrier that allows the activation of the transcription process. It should be noted that all fluorescence changes of the MG/aptamer complex are confined to the microcapsule containments. Centrifuged precipitation of the microcapsules does not lead to any detectable fluorescence of the MG/aptamer complex in the surrounding buffer solution, and the loaded precipitated microcapsules can be resuspended by gentle shaking. Specifically, the introduction of a means to gate the transcription machinery, in bulk buffer solutions, suggests that the incorporation of gated transcription/translation machinery into protocell containments could enable the triggered programmed synthesis of target proteins in analogy to natural processes.

## Conclusions

The study introduced an artificial topological means to switch the transcription of RNA. Three different topological barriers to switch the transcription reaction were introduced including Sr^2+^-ion stabilized G-quadruplex units, T-A·T triplex structures and photoisomerizable azobenzene-nucleic acid blockers. Other topologically triggered reconfigurable nucleic acid structures, such as redox-triggered ligand/aptamer complexes, enzyme-triggered blocker units consisting of aptamer–ligand complexes or enzyme/DNAzyme nicked duplex blockers could provide a means to switch the transcription processes. In addition, in order to mimic native regulation transcription processes, we introduce synthetic transcription machineries and gated transcription machineries that could lead to dose-controlled and programmed gene expression. The artificial model systems mimicking native topologically modulated transcription machinery introduce a dynamic means to yield programmed and modulated RNAs for possible therapeutic applications. Furthermore, primary attempts to integrate the topologically triggered transcription machinery into cell-mimicking containment microcapsules were provided. While the machinery advances systems chemistry by establishing control over gene expression using supramolecular complexes, important challenges are ahead of us. In particular, the significance of the present results rests on the incorporation of the switchable transcription machinery into artificial cell containments. The co-addition of dynamic networks activating the transcription machinery in cell-like containments or the coupling of the transcription machinery to co-added translation machinery in cell-like containments could be important future steps to design synthetic cell-like containments mimicking native systems.

## Author contributions

P. Z., A. F. and I. W. formulated the concepts and methodology of the study. The manuscript was written through the contributions of all authors. All authors participated in the experimental work and have given approval regarding the final version of the manuscript.

## Conflicts of interest

There are no conflicts to declare.

## Supplementary Material

SC-013-D2SC01599D-s001
