# Supplementary material for: Topologically switchable and gated transcription machinery
Source: Chem Sci. 2022 Aug 18;13(35):10555–65. doi: 10.1039/d2sc01599d (PMC9473513; doi:10.1039/d2sc01599d)
Supplement: SC-013-D2SC01599D-s001 [file SC-013-D2SC01599D-s001.pdf]

Electronic Supplementary Material (ESI) for Chemical Science.
This journal is © The Royal Society of Chemistry 2022

Supporting Information for

Topologically Switchable and Gated Transcription Machineries

*Pu Zhang,[a†] Amit Fischer,[a†] Yu Ouyang,[a] Yang Sung Sohn,[b] Rachel Nechushtai,[b] Junji Zhang,[c] He Tian,[c] Chunhai Fan[d] and Itamar Willner[a]**

[†] These authors contributed equally to this work.

[a] Institute of Chemistry, Center for Nanoscience and Nanotechnology, The Hebrew University of Jerusalem, Jerusalem 91904, Israel.

[b] Institute of Life Science, The Hebrew University of Jerusalem, Jerusalem 91904, Israel.

[c] Key Laboratory for Advanced Materials, School of Chemistry and Molecular Engineering, East China University of Science and Technology, Shanghai, China

[d] School of Chemistry and Chemical Engineering, Frontiers Science Center for Transformative Molecules, and National Center for Translational Medicine, Shanghai Jiao Tong University 200240, Shanghai, China.

E-mail: itamar.willner@mail.huji.ac.il

Materials and Methods

Materials and Instruments

Magnesium chloride, potassium chloride, calcium chloride, sodium carbonate, 2-[4-(2-Hydroxyethyl)piperazin-1-yl]ethanesulfonic acid sodium salt (HEPES), Malachite Green (MG), 3,5-difluoro-4-hydroxybenzylidene imidazolinone (DFHBI), carboxymethyl cellulose (CMC, medium viscosity, D.S. 0.9), polyallylamine hydrochloride (PAH, 58 kDa), ethylenediaminetetraacetic acid disodium salt dihydrate (EDTA), agrose, N, N, N', N'-tetramethy-lethylenediamine (TEMED), ammonium persulfate (APS), acrylamide/bis-acrylamide 40% solution (suitable for electrophoresis, 19:1) and Zn(II)-PPIX were purchased from Sigma-Aldrich. T7 RNA polymerase, transcription reaction buffer and NTPs were purchased from New England Biolabs. DNA oligonucleotides were synthesized and purified by Integrated DNA Technologies Inc. (Coralville, IA). GelRed nucleic acid gel stain was purchased from Invitrogen. Ultrapure water from NANOpure Diamond (Barnstead) source was used throughout the experiments.

A Magellan XHR 400L scanning electron microscope (SEM) and an FV-1000 confocal microscope (Olympus, Japan) were employed to characterize the microcapsules. Flow cytometry (CellStream Analyzer, Merck) was used to evaluate the numbers of the microcapsules in the reaction volume. Fluorescence spectra was measured with a Cary Eclipse Fluorometer (Varian Inc.). MG (λ_{ex} = 560 nm, λ_{em} = 665 nm), DFHBI (λ_{ex} = 447 nm, λ_{em} = 500 nm), Cy5 (λ_{ex} = 648 nm, λ_{em} = 668 nm), Zn(II)-PPIX (λ_{ex} = 420 nm, λ_{em} = 550-700 nm). The concentration of DNA oligonucleotides and quantification of T7 RNA polymerase loaded in the microcapsule assemblies were standardized by UV-2401PC (SHIMADAZU) according to Beer-Lambert's Law. The gel experiment was run on a Hoefer SE 600 electrophoresis unit.

The sequences of all nucleic acid used in this study is listed as follows: (from 5' to 3'):

(1):5'-GGATCCATTCGTTACCTGGCTCTCGCCAGTCGGGATGGGTAGGGCGGGTTG GGCCTATAGTGAGTCGTATTAAGCT-3'

(2): 5'-AGCTTAATACGACTCACTATAGG-3'

(3): 5'-GGAGCTCACACTCTACTCAACAGTAGCGAACTACTGGACCCGTCCTTCACC TTTTTTCTTTTCTTTGTTTCTTTCTTTTCTTTTTTCCTATAGTGAGTCGTATTAAGCT -3'

(4): 5'-GGATCCATTCGTTACCTGGCTCTCGCCAGTCGGGATTTTTTCTTTTCTTTGT TTCTTTCTTTTCTTTTTTCCTATAGTGAGTCGTATTAAGCT-3'

(5): 5'-GGATCCATTCGTTACCTGGCTCTCGCCAGTCGGGATATCACTTGATAGAAA CCTATAGTGAGTCGTATTAAGCT-3'

(6): 5'-CCTTTTCTTAGGAGGCTACCTTTTCT azo (CT azo AT azo C A azo AG azo TG azo AT)-3'

(7): 5'-AGAAAAGGTAGCCTCCTAAGAAAAGG-3'

(X): 5'-CTACAT AAA AAA GAA AAGAAA-3'

(X'): 5'-TTT CTT TTC TTT TTT ATG TAG AGC-3'

(X''): 5'-GCTCTACAT AAA AAA GAA AAG AAA-3'

S1: 5'-ACACTACGTCAGAACAGCTTGCATCACTGGTCACCAGAGTAAAAAGCTTA ATACGACTCACTATAGG-3'

S_1-Cy5: 5'-Cy5-ACACTACGTCAGAACAGCTTGCATCACTGGTCACCAGAGTAAAA

AGCTTAATACGACTCACTATAGG-3'

S2: 5'-ACGAGCGAGTTGATGTGATGCAAGCTGAATGCGAGGGTCCT-3'

S3: 5'-TCTGACGTAGTGTATGCACAGTGTAGTTAGGACCCTCGCAT-3'

S4: 5'-TCAACTCGCTCGTTACTACACTGTGCAATACTCTGGTGACC-3'

(8): 5'-GTAGAAGAAGGTGTCACAGTT-3'

(9): 5'-NH_2-$(CH_2)_6$-TTTTCTTCATTGTTT-3'

$\mathbf{H_A}$: 5'-NH_2-$(CH_2)_6$-TTTTTTTTTTGGTGTTTAAGTTGGAGAATTGTACTTAAACACCT TCTTCT-3'

$\mathbf{H_B}$: 5'-CAATTCTCCAACTTAAACTAGAAGAAGGTGTTTAAGTTGGGCTCTAACATC GGTCCAA-3'

Tetrahedra T-(2a) is consisted of (S_1) (S_2) (S_3) and (S_4), Cy5-labeled tetrahedra T-(2a) is consisted of (S_1-Cy5) (S_2) (S_3) and (S_4).

Methods

In vitro transcription

The *in vitro* transcription was carried out by different templates in the presence of T7 polymerase and NTPs. As example, the switchable transcription of the MG aptamer using **(1)**/**(2)** template is presented: 1 μM of template **(1)** and 1 μM of T7 promoter **(2)** were incubated in 1 × transcription reaction buffer containing 14 mM $MgCl_2$ at room temperature. After 20 minutes, 0.026 μM of T7 RNA polymerase, 0.015 M of NTPs and 2 μM of MG were added to the mixture. The transcription was allowed to proceed at 37 °C and the time-dependent formation of the MG aptamer was followed spectroscopically. After a fixed time-interval, 0.02

M of Sr^{2+}-ions were introduced into the system to block the transcription process. Subsequently, 0.06 M of KP was added to the system to reactivate the transcription process.

For the triplex-induced switchable transcription process synthesizing the DFHBI-binding aptamer, 1 μM of template **(3)** and 1 μM of T7 promoter **(2)** were added in 1 × transcription reaction buffer containing 14 mM $MgCl_2$ and incubated at room temperature. After 20 minutes, 0.026 μM of T7 RNA polymerase, 0.015 M of NTPs and 4 μM of DFHBI were added to the mixture. The transcription was allowed to proceed at 37 °C and the time-dependent formation of the DFHBI-binding aptamer was followed spectroscopically. After a fixed time-interval, 10 μM of **(X)** was introduced into the system to block the transcription. Subsequently, 15 μM of **(X')** was added to the system to reactivate the transcription process.

For the triplex switchable transcription process, synthesizing the MG aptamer, 1 μM of template **(4)** and 1 μM of T7 promoter **(2)** was added in 1 × transcription reaction buffer containing 14 mM $MgCl_2$ and incubated at room temperature. After 20 minutes, 0.026 μM of T7 RNA polymerase, 0.015 M of NTPs and 2 μM of MG was added to the mixture. The transcription was allowed to proceed at 37 °C and the time-dependent formation of the MG aptamer was followed spectroscopically. After a fixed time-interval, 10 μM of **(X)** was introduced into the system to block the transcription. Subsequently, 15 μM of **(X')** was added to the system to reactivate the transcription process.

For the light-induced switchable transcription machinery synthesizing the MG aptamer, 1 μM of template **(5)** and 1 μM of T7 promoter **(2)** was added in 1 × transcription reaction buffer containing 14 mM $MgCl_2$ in the presence of *cis*-state **(6c)**/**(7),** 1 μM, and incubated at room temperature. (The **(6c)**/**(7)** was prepared by mixing 50 μM of **(7)** and 50 μM of **(6c)** in 1 × transcription reaction buffer containing 14 mM $MgCl_2$, and incubated at 4 °C overnight, yielding the template **(6c)**/**(7)**.) After 20 minutes, 0.026 μM of T7 RNA polymerase, 0.015 M

of NTPs and 2 μM of MG were added to the mixture. The transcription was allowed to proceed at 40 °C and the time-dependent formation of the MG aptamer was followed spectroscopically. After a fixed time-interval, the system was irradiated with light (λ > 410 nm, 10 minutes). Subsequently, the sample was subjected to UV light of λ = 365 nm, 10 minutes, to reactivate the transcription process.

Gated transcription machineries were carried out as follows. 2 μM of the template **(5)** and 2 μM of T7 promoter **(2)** were added in 1 × transcription reaction buffer containing 14 mM $MgCl_2$ in the presence of 2 μM of **(6c)**/**(7)** and incubated at room temperature. 2 μM of template **(3)** and 2 μM of T7 promoter **(2)** were added in 1 × transcription reaction buffer containing 14 mM $MgCl_2$ and incubated at room temperature. After 20 minutes, the above two solutions were mixed to the final concentration of 2 μM of T7 promoter **(2)** and 1 μM of each DNA template, followed by subjecting 0.026 μM of T7 RNA polymerase, 0.03 M of NTPs and 2 μM of MG (or 4 μM of DFHBI) to the mixture. The transcription was allowed to proceed at 40 °C and the time-dependent formation of the MG aptamer or DFHBI-binding aptamer was followed spectroscopically, state "O". After a fixed time-interval, light irradiation (λ > 410 nm, 10 minutes) was subjected to state "O" to yield state "P" where the MG aptamer transcription is blocked. Subsequently, light irradiation (λ=365 nm, 10 minutes) was applied to the system to reactivate the light-induced machinery, regenerating state "O". By subjecting the system with illumination (λ = 410 nm, 10 minutes), the light-induced machinery was re-blocked, re-generating the state "P". The state "Q" was yielded by introducing 10 μM of **(X)** into state "O". Subsequently, 15 μM of **(X')** was added to the system to reactivate the triplex switchable machinery, regenerating state "O". By subjecting the system with 20 μM of **(X'')**, the triplex switchable machinery was re-blocked, re-generating the state "Q".

Construction of DNA tetrahedra nanostructure

The DNA tetrahedra, used in this study, consisted of four sequences, S_1, S_2, S_3 and S_4 or S_1-Cy5, S_2, S_3 and S_4. The synthesis of the tetrahedra followed the steps: A mixture of (S_1) or (S_1-Cy5), (S_2), (S_3) and (S_4) (4 µM each) in 10 mM HEPES buffer (containing 20 mM $MgCl_2$, pH = 7.4) was annealed at 95 °C for 5 min, subsequently, cooled down to 4 °C, and allowed to equilibrate at 25 °C for 2 hours, yielding DNA tetrahedra T-(2a) or Cy5-T-(2a). The assembly of the tetrahedra DNA nanostructures was characterized by native PAGE, Figure S6.

Synthesis of 5'-Amino Modifier C_6-modified oligo (H_A and (9))/Carboxymethyl cellulose (CMC) copolymers

2 mL of a MES buffer solution (10 mM, pH 5.5) containing CMC, 20 mg, N-(3-dimethylaminopropyl)-N'-ethylcarbodiimide hydrochloride (EDC), 20 mg, were incubated for 5 minutes and then sulfo-N-hydroxysuccinimide (NHS), 26 mg, was added, and the solution was incubated and shaken for additional 10 minutes. To the resulting solution, 2 mL of HEPES buffer (50 mM, pH 7.4) containing the amine-functionalized nucleic acids (900 µM of H_A for P_A or 900 µM of (**9**) for P_B) were added. The mixture was gently shaken for 2 hours at room temperature and then left to react at 4°C for additional 12 hours. The modified polymers, P_A (modified with H_A) and P_B (modified with **(9)**), were purified and separated from the unreacted compounds using MWCO 10K Amicon spin filters (5000 rcf, 15 minutes). After being washed with water for five times, the copolymer solutions were dried and re-suspended in 10 mM HEPES buffer containing 25 mM $MgCl_2$, pH 7.4. To polymer P_B, after the determination of the concentration of (**9**), hairpin H_B was added in a molar ratio of 1:1. The polymer solutions were incubated at 95 °C for 5 min, immediately followed by incubation at 0 °C for 30 min to ensure the efficient closure of the hairpins. All polymer samples were kept at 4 °C for further use. The final concentration of P_A (modified with H_A) and P_B were 2 mg/mL and of H_A and H_B were 60 µM.

Preparation of microcapsules loaded with scaffold (1)/T-(2a) and T7 RNA polymerase

4 μM of **(1)** and 4 μM of T-**(2a)** was incubated at room temperature for 2 hours in 10 mM HEPES buffer containing 25 mM $MgCl_2$. $CaCO_3$ microparticles loaded with scaffold **(1)**/T-**(2a)** and T7 RNA polymerase were prepared by mixing $CaCl_2$ (307 μL, 0.33 M) and Na_2CO_3 (307 μL, 0.33 M) solutions with scaffold (1)/T-**(2a)** (60 μL, 4 μM), T7 RNA polymerase (60 μL, 0.8 μM) and H_2O (285 μL). The mixture was stirred for two minutes and the resulted precipitate was collected and washed twice with water (at 100 rcf for 30 s). The resulting precipitate was suspended in 600 μL of 10 mM HEPES buffer containing 25 mM $MgCl_2$, pH 7.4, that included 0.6 mg of PAH and allowed, while shaking to incubate for 30 minutes. The resulting particles were collected and washed twice with the HEPES buffer solution. The PAH-coated microparticles were incubated with 600 μL of the promoter nucleic acid (**8**) (final concentration 16.67 μM) and kept under continuous shaking at room temperature for 30 min. After being washed twice with buffer (10 mM HEPES, pH 7.4, containing 25 mM $MgCl_2$), followed by centrifugation at 100 rcf for 30 s, the DNA hydrogel particles were prepared by mixing the polymer chains (P_A/P_B, 20 μL of each, 2mg/mL of the polymer solutions) with the promoter-coated $CaCO_3$ microparticles. The particles were incubated overnight (approximately 12 hours) at room temperature under continuous shaking, followed by centrifugation at 100 rcf for 30 s to remove non-adsorbed polymers and subsequently resuspended in 200 μL buffer (10 mM HEPES, pH 7.4, containing 25 mM $MgCl_2$). This washing procedure was repeated twice. Subsequently, 60 μL of microparticle solution was introduced into 50 μL of 0.5 M EDTA solution (pH 8) for 0.5 h to dissolve the $CaCO_3$ cores and to yield the hydrogel microcapsules. When the suspension became clear, the capsules were washed with buffer (10 mM HEPES, pH 7.4, containing 25 mM $MgCl_2$) for three times (50 rcf, 20 min), and resuspended in 100 μL of the buffer. The concentration of the microcapsules was evaluated by flow cytometry and it corresponded to 4500 microcapsules/μL.

Switchable transcription of the MG aptamer in microcapsule protocell assemblies

To explore the switchable transcription process of protocells that loaded with **(1)**/T-**(2a)** and T7 RNA polymerase (depicted in Figure 5 (A)), 2 μM of MG and 0.05 M of NTPs were added to 25 μL of the etched microcapsule protocell solution (4500 microcapsules/μL) in the presence of 1 × transcription reaction buffer. The transcription in microcapsule protocell assemblies was allowed to proceed at 30 °C and was probed by following the time-dependent formation of the fluorescence of the MG/aptamer complex, λ_{ex} = 560 nm, λ_{em} = 665 nm. After a fixed time-interval, Sr^{2+}-ions, 0.02 M, were added to system to block the transcription process. Subsequently, KP, 0.06 M, at the appropriate time-interval, was added to the system to reactivate the transcription process. Finally, Sr^{2+}-ions, 0.08 M, are re-added to the system to re-block the transcription machinery.

Confocal microscopy imaging of the transcription process in the microcapsules

5 μL of microcapsule samples (4500 microcapsules/μL) were loaded on a microscopic glass slide protected with coverslips. 2 μM of MG and 0.05 M of NTPs were added to one of the first samples, and bright field and the fluorescence microscopy images of the sample were recorded at time=0 and time=24 h, Figure 6 (C), panel I and II. A second sample was treated with 2 μM of MG and 0.02 M of Sr^{2+}-ions, after 30 minutes, 0.05 M of NTPs were added to the system, and the bright field and fluorescence microscopy images were recorded after 24 hours, Figure 6 (C), panel III. The third sample was treated with 2 μM of MG and 0.02 M of Sr^{2+}-ions, after 24 hours, 0.06 M of KP and 0.05 M of NTPs were added to the system. The bright field and fluorescence microscopy images were recorded after 24 hours, Figure 6 (C), panel IV.

Fluorescence images of the microcapsules were recorded with a confocal microscope (Olympus FV3000 confocal laser-scanning microscope), MG channel: E_x=635 nm, E_m=650-700 nm, and images were analyzed using the Image J software.

Characterization of Sr^{2+}-ion stabilized G-quadruplex embedded in scaffold (1)

Two samples of **(1)**, 2 μM, and Zn(II)-PPIX, 2 μM in 10 mM HEPES buffer containing 14 mM $MgCl_2$, 100 μL, were prepared. To one sample, 0.02 M of Sr^{2+}-ions were added to induce the generation of the Sr^{2+}-ion -stabilized G-quadruplex unit. Figure S1, curve (a) depicts the intense fluorescence spectrum of Zn(II)-PPIX associated with the Sr^{2+}-ion stabilized G-quadruplex. The sample of **(1)** in the presence of Zn(II)-PPIX, yet in the absence of Sr^{2+}-ions shows a very weak fluorescence spectrum, Figure S1, curve (b), indicating that in the absence of Sr^{2+}-ions, no G-quadruplex is formed. (*Ref.* Zhang, Z.; Sharon, E.; Freeman, R.; Liu, X.; Willner, I., Fluorescence Detection of DNA, Adenosine-5'-Triphosphate (ATP), and Telomerase Activity by Zinc(II)-Protoporphyrin IX/G-Quadruplex Labels. *Anal. Chem.* **2012,** *84*, 4789-4797.)

The Sr^{2+}-ions-induced formation of the Sr^{2+}-ion -stabilized G-quadruplex embedded in **(1)** is further supported by the intensified circular dichroism spectra of **(1)** upon addition of Sr^{2+}-ions, *cf.* Figure S2, curve (b) *vs.* (a).

Evaluation of the loadings of the Cy5-labeled (1)/T-(2a) scaffold and T7 RNA polymerase in the microcapsules

$CaCO_3$ microparticles loaded with scaffold Cy5-labeled **(1)**/T-**(2a)** and T7 RNA polymerase were prepared by mixing $CaCl_2$ (307 μL, 0.33 M) and Na_2CO_3 (307 μL, 0.33 M) solutions with scaffold Cy5-labeled **(1)**/T-**(2a)** (60 μL, 4 μM), T7 RNA polymerase (60 μL, 0.8 μM) and H_2O (285 μL). The mixture was stirred for two minutes at room temperature and the particles were

precipitated by centrifugation 100 rcf for 30 s. The precipitate was washed with water twice (100 rcf for 30s). The upper solution was collected. Subsequently, the resulting precipitate was suspended in 600 μL of 10 mM HEPES buffer containing 25 mM $MgCl_2$, pH 7.4, that included 0.6 mg of PAH and allowed to shake for 30 minutes. The resulting particles were collected and washed twice with the HEPES buffer solution. The PAH-coated microparticles were incubated with 600 μL of the promoter nucleic acid (**8**) (final concentration 10 μM) and kept under continuous shaking at room temperature for 30 min. After being washed twice with buffer (10 mM HEPES, pH 7.4, containing 25 mM $MgCl_2$), followed by centrifugation at 100 rcf for 30 s. 20 μL of each of the P_A and P_B polymer solutions (2mg/mL) were added to the promoter-coated $CaCO_3$ microparticles in a final volume of 600 μL HEPES buffer, and the HCR process generated the hydrogel coating on the particles. The particles were incubated overnight (approximately 12h) at room temperature under continuous shaking, followed by centrifugation at 100 rcf for 30 s to remove non-adsorbed polymers and subsequently were resuspended in buffer (10 mM HEPES, pH 7.4, containing 25 mM $MgCl_2$). This washing procedure was repeated twice. Subsequently, 60 μL of microparticle suspension were introduced into 120 μL of 0.5 M EDTA solution (pH 7.5) for 0.5 h to dissolve the $CaCO_3$ cores and to yield the hydrogel microcapsules. The suspension became clear, the capsules were washed with buffer (10 mM HEPES, pH 7.4, containing 25 mM $MgCl_2$) for three times (50 rcf, 20 min) and the upper solution was collected.

The loading of Cy5-labeled **(1)**/T-**(2a)** in the resulting microcapsules was evaluated by two methods: (i) The fluorescence spectrum of the Cy5-labeled **(1)**/T-**(2a)** and T7 RNA polymerase-loaded microcapsules was recorded, Figure S4 (A), and by using the appropriate calibration curve, the total concentration of the entrapped Cy5-labeled **(1)**/T-**(2a)** was evaluated to be 2.7×10^{-11} moles in the overall mixture of capsules. Knowing the number of the microcapsules (4500 microcapsules/μL), an average loading of 2.9×10^{-17} moles per capsule

was estimated. (ii) We find that a low content of the Cy5-labeled **(1)**/T-**(2a)** was not adsorbed in the $CaCO_3$ microparticles in the first step of particle impregnation. In addition, we find that upon etching the hydrogel coated $CaCO_3$ microparticles, a certain content of Cy5-labeled **(1)**/T-**(2a)** is released to the bulk solution. The contents of non-adsorbed Cy5-labeled **(1)**/T-**(2a)** in the primary step of particles formation and the content of released Cy5-labeled **(1)**/T-**(2a)** upon etching the particles, Figure S4 (B), were evaluated. Knowing the initial content of Cy5-labeled **(1)**/T-**(2a)** and appropriate subtraction of the content of the scaffold during the preparation of the microcapsules, we estimate that 2.4×10^{-10} moles of Cy5-labeled **(1)**/T-**(2a)** was entrapped in the microcapsules (2.6×10^{-16} moles per capsule), in good agreement of the value evaluated by method (i).

Realizing that the enzyme exhibits an absorbance in the region 250-300 nm, the loading of T7 RNA polymerase was evaluated spectroscopically. The loading was estimated as follows: (a) The content of T7 RNA polymerase adsorbed onto the $CaCO_3$ microparticles was determined by evaluation of the content of the non-adsorbed enzyme in the solution upon preparation of the particle and subtracting of this content from the parent content of enzyme used to prepare the particles. (b) We find that upon etching of the particles residual amount of the enzyme are released into the bulk solution. Figure S4 (C), curve A1 depicts the absorbance spectrum of the bulk solution, generated upon etching of the hydrogel coated particles, in the region 250-300 nm. Realizing that DNA has a residual absorbance in this region, the band A1 represents the combined absorbance of T7 RNA polymerase and the Cy5-labeled **(1)**/T-**(2a)**. Using the absorbance spectrum (A2) of known concentration T7 RNA polymerase (0.008 μM), the amount of T7 RNA polymerase in the bulk solution was evaluated. Realizing the content of Cy5-labeled **(1)**/T-**(2a)**, in the bulk solution, was evaluated independently by fluorescence spectroscopy, we subtracted the estimated absorbance contribution of the DNA to the band A1 to yield the net absorbance of T7 RNA polymerase. Using appropriate calibration curve, the

estimated total concentration of T7 RNA polymerase in the microcapsules corresponding to 1.11×10^{-11} moles in the overall mixture of capsules (or 1.23×10^{-17} moles per capsule). In fact, the results indicate that > 85% of the adsorbed T7 RNA polymerase in the primary step of preparation of the particle retain as encapsulated biocatalyst in the microcapsules.

Electrophoretic characterization of the tetrahedra T-(2a) and electrophoretic characterization of (1)/T-(2a) and the effect of EDTA upon etching the $CaCO_3$ core

The intact individual constituents S_1, S_1+S_2, S_1+S_2+S_3 and S_1+S_2+S_3+S_4 at identical concentration, 1.0 μM, were loaded in predefined lanes in the presence of 1 × loading dye. The separated constituents were stained with GelRed. Native PAGE gel electrophoresis experiments were performed using an acrylamide gel (7%, acrylamide/bis acrylamide 19:1), gel thickness, 1 mm. Mixtures were separated upon applying a 100 V potential, 4 °C (to eliminate the dissociation of duplexes). The separation was conducted for a time-interval of 16 hours. Figure S6 indicates the step-wised synthesis of tetrahedra T-**(2a)**.

$CaCO_3$ microparticles loaded with scaffold **(1)**/T-**(2a)** were prepared by mixing $CaCl_2$ (307 μL, 0.33 M) and Na_2CO_3 (307 μL, 0.33 M) solutions with scaffold **(1)**/T-**(2a)** (60 μL, 4 μM) and H_2O (285 μL). The mixture was stirred for two minutes at room temperature and was precipitated by 100 rcf for 30 s. The precipitate was washed with water twice (100 rcf for 30s). The resulting precipitate was suspended in 600 μL of 10 mM HEPES buffer containing 25 mM $MgCl_2$, pH 7.4, and allowed to shake for 30 minutes. Subsequently, 10 μL of the resulting microparticle solution (22500 microcapsules/μL) was introduced into 20 μL of 0.5 M EDTA solution (pH 8) for 0.5 h to dissolve the $CaCO_3$ cores and to yield the etched **(1)**/T-**(2a)** structure. The etched sample was subjected to 1 × loading dye for the electrophoretic characterization. As comparison, the parent 1 μM of **(1)**/T-**(2a)**, as reference, was subjected to 1 × loading dye to demonstrate the intact structure of etched **(1)**/T-**(2a)**. Electrophoretic

separation was carried out on 2% agarose gel, 100 V, 4 °C. The separation was conducted for a time-interval of 1.5 hours. The gel was stained with GelRed. We find that the tetrahedra after the etching process did not show any side-band of separated products. These results from Figure S7 support the intact structure of the **(1)**/T-**(2a)** template in the microcapsule assembly for the respective transcription process.

Leakage of the Cy5-labeled (1)/T-(2a) scaffold in the microcapsule assemblies

25 μL of microcapsules loaded with scaffold Cy5-labeled **(1)**/T-**(2a)** and T7 RNA polymerase were used to evaluate the leakage of Cy5-labeled **(1)**/T-**(2a)** scaffold from the microcapsules. 25 μL of microcapsules was centrifuged at 50 rcf for 20 min and resuspend in 120 μL of 10 mM HEPES buffer containing 25 mM $MgCl_2$, pH 7.4. After that, the suspension microcapsules and the washing solution were measured on a fluorescence meter, respectively, Figure S5. No fluorescence of the Cy5-labeled **(1)**/T-**(2a)** in the bulk solution could be detected within a time-interval of three days indicating the stable confinement of the transcription template in the microcapsule system.

Evaluation of the activity of the T7 RNA polymerase after $CaCO_3$ impregnation

$CaCO_3$ microparticles loaded with T7 RNA polymerase were prepared by mixing $CaCl_2$ (307 μL, 0.33 M) and Na_2CO_3 (307 μL, 0.33 M) solutions with T7 RNA polymerase (60 μL, 0.8 μM) and H_2O (345 μL). The mixture was stirred for two minutes and the resulted precipitate was collected and washed twice with water (at 100 rcf for 30 s). The resulting precipitate was suspended in 600 μL of 10 mM HEPES buffer containing 25 mM $MgCl_2$, pH 7.4, and allowed, while shaking to incubate for 30 minutes. The quantification of the T7 RNA polymerase loaded in the $CaCO_3$ microparticles was evaluated by measuring the absorbance of the collected washing solution and was estimated to be 2.7×10^{-10} moles in the overall volume of capsules

(200 μL). Subsequently, 20 μL of the resulting microparticle solution was introduced into 40 μL of 0.5 M EDTA solution (pH 8) for 0.5 h to dissolve the $CaCO_3$ cores and to yield the etched T7 RNA polymerase. 2 μM of scaffold **(1)**/T-**(2a)**, 0.05 M of NTPs, 2 μM of MG was introduced to the resulting solution in the presence of 1 × transcription reaction buffer. For comparison, by using the identical quantity of the T7 RNA polymerase in microparticles, 2.7 × 10^{-11} moles of T7 RNA polymerase was subjected to 120 μL buffer solution containing 2 μM of scaffold **(1)**/T-**(2a)**, 0.05 M of NTPs, 2 μM of MG to proceed the transcription process. Figure S8 shows the activities of similar contents of T7 RNA polymerase without immobilization of $CaCO_3$ and subsequent EDTA etching, curve (a), and of T7 RNA polymerase deposited in $CaCO_3$ and etched with EDTA, curve (b). The results indicate that upon impregnation of T7 RNA polymerase on $CaCO_3$ and subsequent EDTA etching, the enzyme loses *ca.* 30% of its activity.

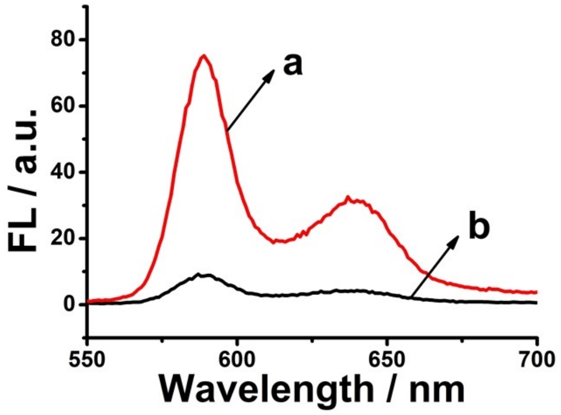

Figure S1 Fluorescence spectra corresponding to: (a) scaffold **(1)**, 2 μM, and Zn(II)-PPIX, 2 μM, in the presence of 0.02 M of Sr^{2+}-ions. (b) scaffold **(1)**, 2 μM, and Zn(II)-PPIX, 2 μM, in the absence of 0.02 M of Sr^{2+}-ions.

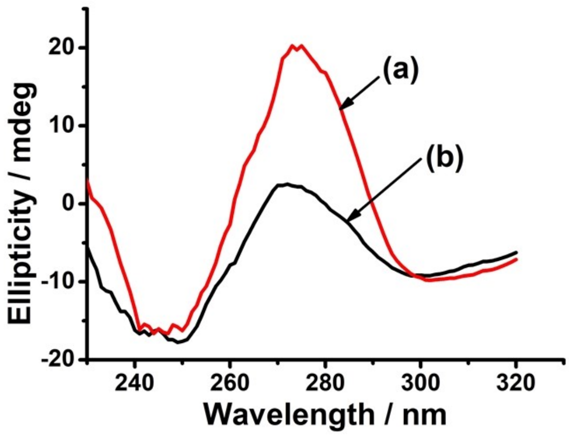

Figure S2 Circular dichroism spectra corresponding to: (a) Scaffold **(1)**, 2 μM, upon addition of Sr^{2+}-ions, 0.02 M. (b) Scaffold **(1)**, 2 μM. The intensified circular dichroism spectrum is consistent with the formation of the Sr^{2+}-ions stabilized G-quadruplex.

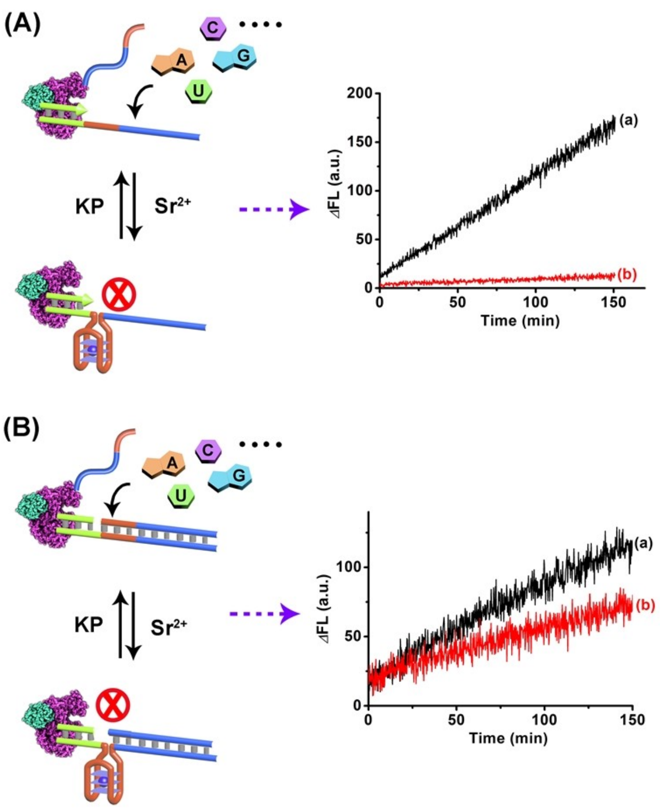

Figure S3 (A) Schematic Sr^{2+}-ions (0.02 M)/kryptofix [2.2.2], KP (0.06 M), topologically switchable transcription of the MG aptamer upon the reconfiguration of single-stranded template in the presence of T7 RNA polymerase, NTPs and MG. And time-dependent fluorescence changes upon the switchable transcription of the MG/aptamer complex by the transcription machinery. (a) In the absence of KP. (b) In the presence of strand KP, 0.06 M. (B) Schematic Sr^{2+}-ions (0.02 M)/kryptofix [2.2.2], KP (0.06 M), topologically switchable transcription of the MG aptamer upon the reconfiguration of duplex template in the presence of T7 RNA polymerase, NTPs and MG. And time-dependent fluorescence changes upon the switchable transcription of the MG/aptamer complex by the transcription machinery. (a) In the absence of KP. (b) In the presence of strand KP, 0.06 M.

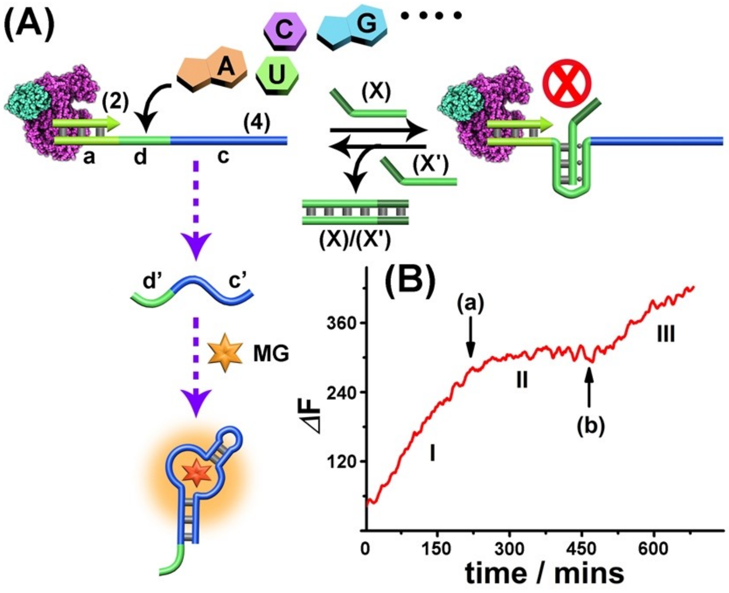

Figure S4 (A) Schematic T-A·T triplex topologically switchable transcription of the MG aptamer in the presence of T7 RNA polymerase, NTPs and MG. The strand **(X)** yields the triplex structure to switch-OFF the transcription process, whereas the displacement of **(X)** by the strand **(X')** switches-ON the transcription machinery. (B) Time-dependent fluorescence changes upon the switchable transcription of the MG/aptamer complex by the transcription machinery. (I) In the absence of strand **(X)**. (II) In the presence of strand **(X)**, 10 µM, introduced at time marked (i). (III) At time marked (ii), **(X')**, 15 µM, is added to the system.

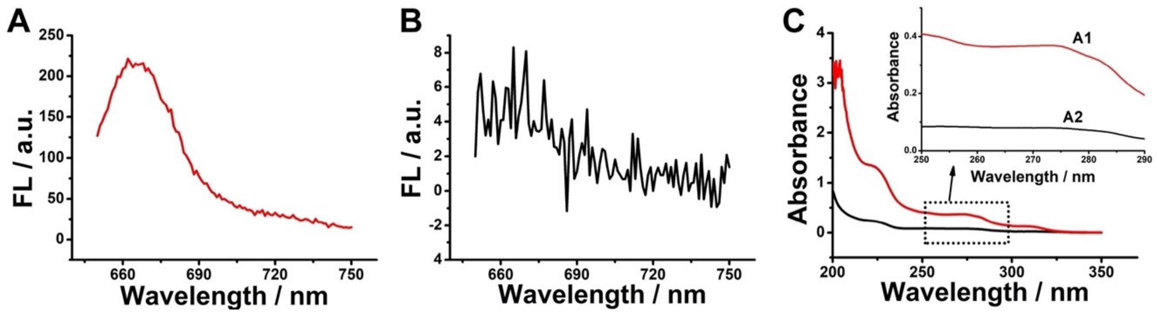

Figure S5 (A) The fluorescence spectrum of the Cy5-labeled **(1)**/T-**(2a)** and T7 RNA polymerase-loaded microcapsules. (B) The fluorescence spectrum of the supernatant generated from the microcapsules loaded with Cy5-labeled **(1)**/T-**(2a)** and T7 RNA polymerase. (C) (A1) The absorption spectrum of the supernatant from the precipitated microcapsules loaded with Cy5-labeled **(1)**/T-**(2a)** and T7 RNA polymerase. (A2) The absorption spectrum of 0.008 μM of T7 RNA polymerase.

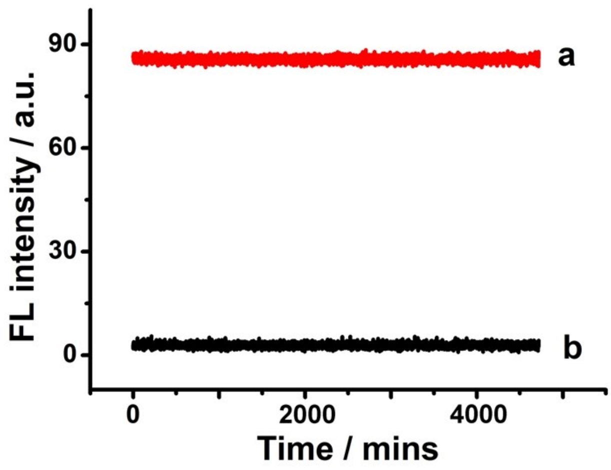

Figure S6 Time-dependent fluorescence intensity of: (a) Cy5-labeled **(1)**/T-**(2a)**-loaded microcapsular protocell, and (b) the supernatant of the Cy5-labeled **(1)**/T-**(2a)**-loaded microcapsular protocell. No leakage of the Cy5-labeled **(1)**/T-**(2a)** from the microcapsule containments could be detected within this time-interval.

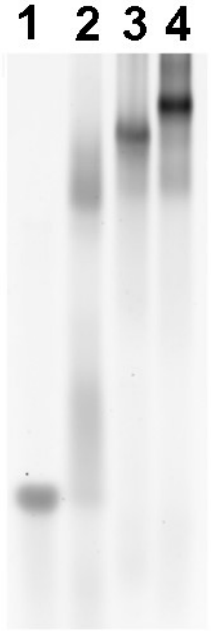

Figure S7 Native polyacrylamide gel electrophoresis (PAGE) analysis of the formation of the tetrahedra T-**(2a)**: Lane 1-S_1 (1 μM); Lane 2-S_1+S_2 (1 μM of each strand); Lane 3- S_1+S_2+S_3 (1 μM of each strand); Lane 4-S_1+S_2+S_3+S_4 (1 μM of each strand).

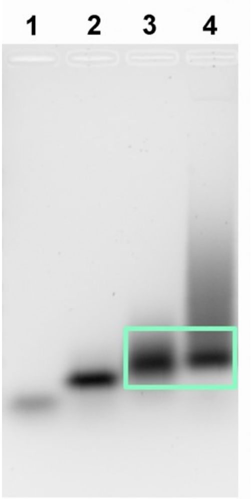

Figure S8 Electrophoretic characterization of the **(1)**/T-**(2a)** and effect of EDTA upon etching the $CaCO_3$ core on which the **(1)**/T-**(2a)** nanostructures are deposited. (Electrophoretic separation on 2% agarose gel, 100 V, 1.5h). Lane 1-Scaffold **(1)**; Lane 2-T-**(2a)**; Lane 3-Intact structure of **(1)**/T-**(2a)**; Lane 4-Intact tetrahedra (with no fragmented strands) upon analysis of the solution obtained after EDTA etching, 0.1 M, of the $CaCO_3$ particles impregnated with the **(1)**/T-**(2a)**. The results demonstrated that the deposition of **(1)**/T-**(2a)** on the $CaCO_3$ core and the EDTA etching had no effect on the intact structure of **(1)**/T-**(2a)**.

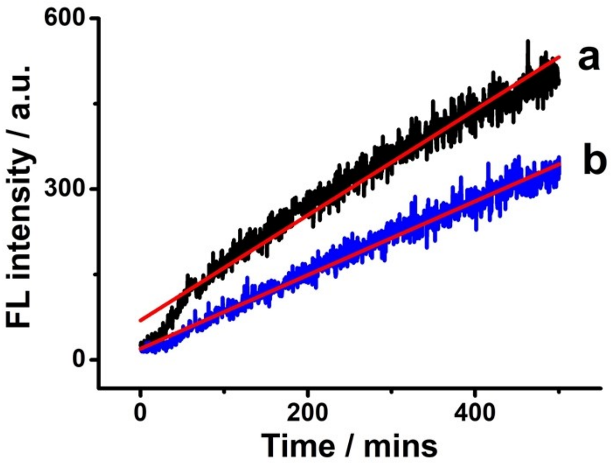

Figure S9 Time-dependent fluorescence changes upon the transcription of the MG aptamer by (a) T7 RNA polymerase without immobilization of $CaCO_3$ and (b) subsequent EDTA etching, in the presence of 2 μM of scaffold **(1)**/T-**(2a)**, 0.05 M of NTPs, 2 μM of MG.

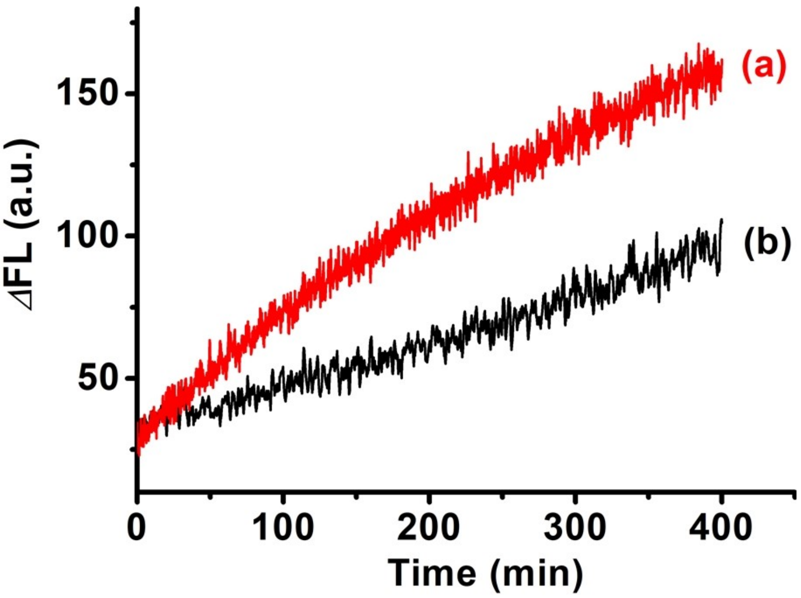

Figure S10 Time-dependent fluorescence changes upon the transcription of the MG aptamer by (a) the homogeneous buffer solution, where the tetrahedra template and T7 polymerase are present at identical concentrations present in microcapsules, and (b) the suspension of the loaded microcapsules in the presence of 2 μM of MG and 0.05 M of NTPs.
